# Application of Ice for Postoperative Total Knee Incisions – Does this Make Sense? A Pilot Evaluation of Blood Flow Using Fluorescence Angiography

**DOI:** 10.7759/cureus.5126

**Published:** 2019-07-11

**Authors:** Devon Foster, Jeb Williams, Antonio J Forte, Elizabeth R Lesser, Michael G Heckman, Glenn G Shi, Joseph L Whalen, Benjamin K Wilke

**Affiliations:** 1 Miscellaneous, Mayo Clinic, Jacksonville, USA; 2 Orthopedics, Mayo Clinic, Jacksonville, USA; 3 Division of Plastic Surgery and Robert D. and Patricia E. Kern Center for the Science of Health Care Delivery, Mayo Clinic, Jacksonville, USA

**Keywords:** total knee arthroplasty, fluorescence angiography, cryotherapy, pain, blood flow

## Abstract

Introduction

Total knee arthroplasty (TKA) is a common procedure with significant advances over the past several years, many pertaining to improved perioperative pain control. Cryotherapy is one method thought to decrease swelling and pain postoperatively. To our knowledge no study has directly visualized the effect cryotherapy has on skin blood flow following TKA. The primary aim was to determine if cryotherapy (icing) affects peri-incisional skin blood flow and if this is lessened with an alternate placement of the ice. We hypothesized that blood flow would decrease following cryotherapy, and this decrease would be greater with ice placed directly over the incision as compared to placement along the posterior knee.

Methods

This study included 10 patients who underwent TKA. During the postoperative hospitalization, they were given an injection of indocyanine green dye. A baseline image was recorded of the skin blood flow. Images were then collected following a five-minute interval placement of ice over the incision. The experiment was then repeated with the ice placed along the posterior knee.

Results

There was an approximate 40% decrease in skin blood flow following placement of the ice compared to baseline. We observed a greater decrease in blood flow when ice was placed over the incision as compared to when ice was placed posterior to the knee (*p* ≤ 0.020).

Conclusion

We found a significant decrease in peri-incisional blood flow with icing of the knee. Physicians should be cognizant of this when recommending cryotherapy to patients after surgery, especially in at-risk wounds.

## Introduction

Total knee arthroplasty (TKA) is one of the most common orthopedic procedures performed in the United States [1]. Many of the advances in total knee arthroplasty over the past several years have been in improved perioperative management. Recent efforts have particularly focused on enhanced alternative pain control methods to decrease reliance on opioid narcotics. These efforts include greater utilization of non-narcotic medications such as non-steroidal anti-inflammatories and steroids, as well as cryotherapy [2]. It is believed that cryotherapy will reduce inflammation, swelling, and pain, and be overall beneficial for recovery [2-3]. This has prompted several companies to produce cooling devices advertised for use after injury or surgery. However, studies also have demonstrated decreased blood flow to the skin once cooled, theoretically increasing the risk of wound complications [4-5]. These previous studies have evaluated skin blood flow using indirect means of measurement. To our knowledge, no study has directly visualized the effect cooling has on the skin blood flow around a postoperative total knee incision.

In the current study, we visualize the cutaneous blood flow using laser-induced fluorescence angiography with indocyanine green (ICG) dye both at baseline and following placement of ice over the incision as well as on the posterior aspect of the knee. ICG is a dye that binds to plasma proteins and is confined to the vascular circulation [6]. The dye has been used extensively in plastic surgery, general surgery, and now orthopedics to measure skin blood flow [7-9]. 

The primary aim was to determine if our current method of applying ice directly to the incision postoperatively affects peri-incisional skin blood flow, and if any reduction observed can be lessened by placing the ice in alternate locations. We hypothesized that using ice would cause a decrease in the observed skin blood flow, and this decrease would be greater when the ice was placed directly over the incision as compared to the posterior placement.

## Materials and methods

Study subjects

Following institutional review board approval, we conducted a prospective evaluation of peri-incisional skin blood flow around a postoperative total knee arthroplasty at baseline and following application of ice to determine the effect cooling has on peri-incisional skin blood flow. Incisions were closed with a 3-0 monofilament subcuticular closure with steri-strips or a prineo on the skin, per the treating surgeon’s preference.

Patients were eligible for the study if they underwent primary total knee arthroplasty. They were excluded if they had an allergy to the ICG dye, if they underwent a revision procedure rather than a primary joint replacement, or if they had previous incisions around the knee (other than arthroscopy portals). A total of 10 patients were included in this study.

Blood flow assessment

Laser-induced fluorescence angiography with indocyanine green dye was used to visualize and record the skin blood flow. During the postoperative hospital stay, the dressing was removed on either postoperative day one or two, per the treating surgeon’s preference. After removal of the dressing, the patient was given a 5-mL intravenous injection of ICG dye by the nursing staff followed by a 10-mL flush. Three minutes were timed before collection of the imaging to allow the dye to circulate to the knee. A baseline image was collected using the PDE NEO II fluorescence imager (Mitaka - Denver, CO). The camera was held 6 inches from the skin, centering the incision in the field of view for the baseline image acquisition as well as all subsequent imaging to maintain consistency.

Following the collection of the baseline image, a bag containing ice was placed over the incision for five minutes (anterior, or incisional icing). After five minutes, a second image was obtained. Images were then captured every 30 seconds for an additional two minutes.

Subsequently, the skin temperature was allowed to normalize, and an additional injection of ICG dye was given followed by a three-minute interval to allow the dye to equilibrate before obtaining a new baseline image of the incision. The ice bag was then placed on the posterior aspect of the knee and kept in place for five minutes, and after this five-minute period, a second image was obtained. Additional imaging of the incision was performed similarly as was used for the anterior or incisional icing (every 30 seconds for two minutes).

ImageJ (NIH) was used to evaluate the images obtained. A standard 400 x 280-pixel box centered over the incision was drawn to capture the mean signal intensity in the tissue around the incision. The baseline images for both the incisional and posterior icing were standardized to 100% with subsequent images reported as a ratio of the respective baseline image.

Statistical analysis

Continuous variables were summarized using the median and range while sex and laterality of the operated knee were summarized using frequency and percent. Separately for incisional and posterior areas, blood flow at each time point was measured by calculating the percent change from baseline for each patient. To assess differences in blood flow in comparison to the baseline time point, we compared these percent changes from baseline at the post-baseline time points to a value of zero using a one-sample Wilcoxon rank sum test, separately for the incisional and posterior areas. To compare changes from baseline at post-baseline time points between incisional and posterior areas, we used a paired Wilcoxon signed rank test. 

We also assessed percent changes in blood flow between sequential time points (i.e., 0 to 30 seconds, 30 to 60 seconds, etc.). These changes were compared to a value of zero using a one-sample Wilcoxon rank sum test separately for incisional and posterior areas and were compared between incisional and posterior areas using a paired Wilcoxon signed rank test. All tests were two-sided and performed at the 0.05 significance level. All statistical analyses were performed in R Statistical Software (version 3.4.2; R Foundation for Statistical Computing, Vienna, Austria).

## Results

This study included four males (40%) and six females (60%). Their overall median age was 58 years (range: 51 to 72). An equal number of TKAs were performed on the left and right side. The median body mass index (BMI) was 32.9 (range: 28.6 to 48.4). All patients were followed postoperatively, and there were no wound complications.

**Figure 1:**
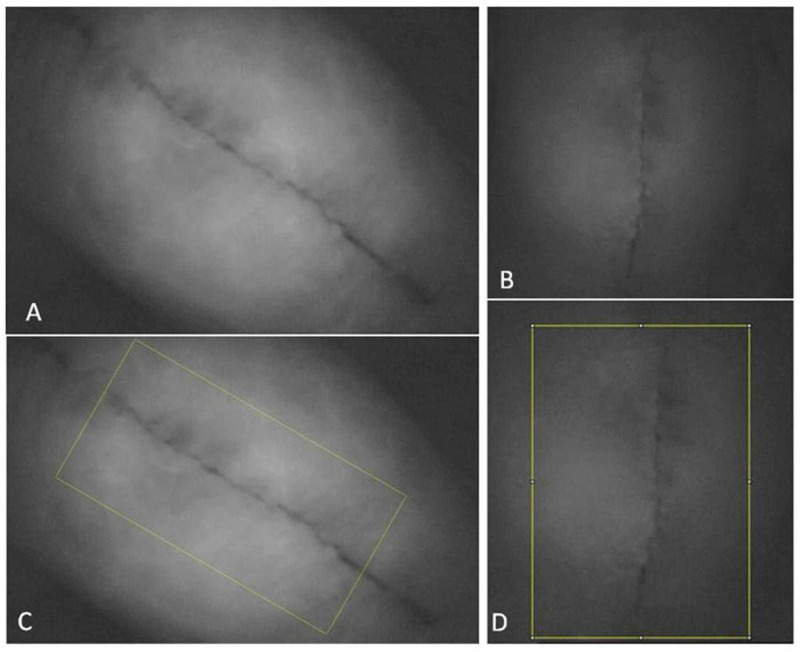
A baseline image is shown in (A), followed by an image captured immediately after the ice was removed from the anterior (incisional) knee (B). The ImageJ capture points are shown in (C) and (D) for the respective photos The indocyanine green dye within the blood vessels shows up as white on the images, while dark areas are devoid of blood flow.

**Figure 2:**
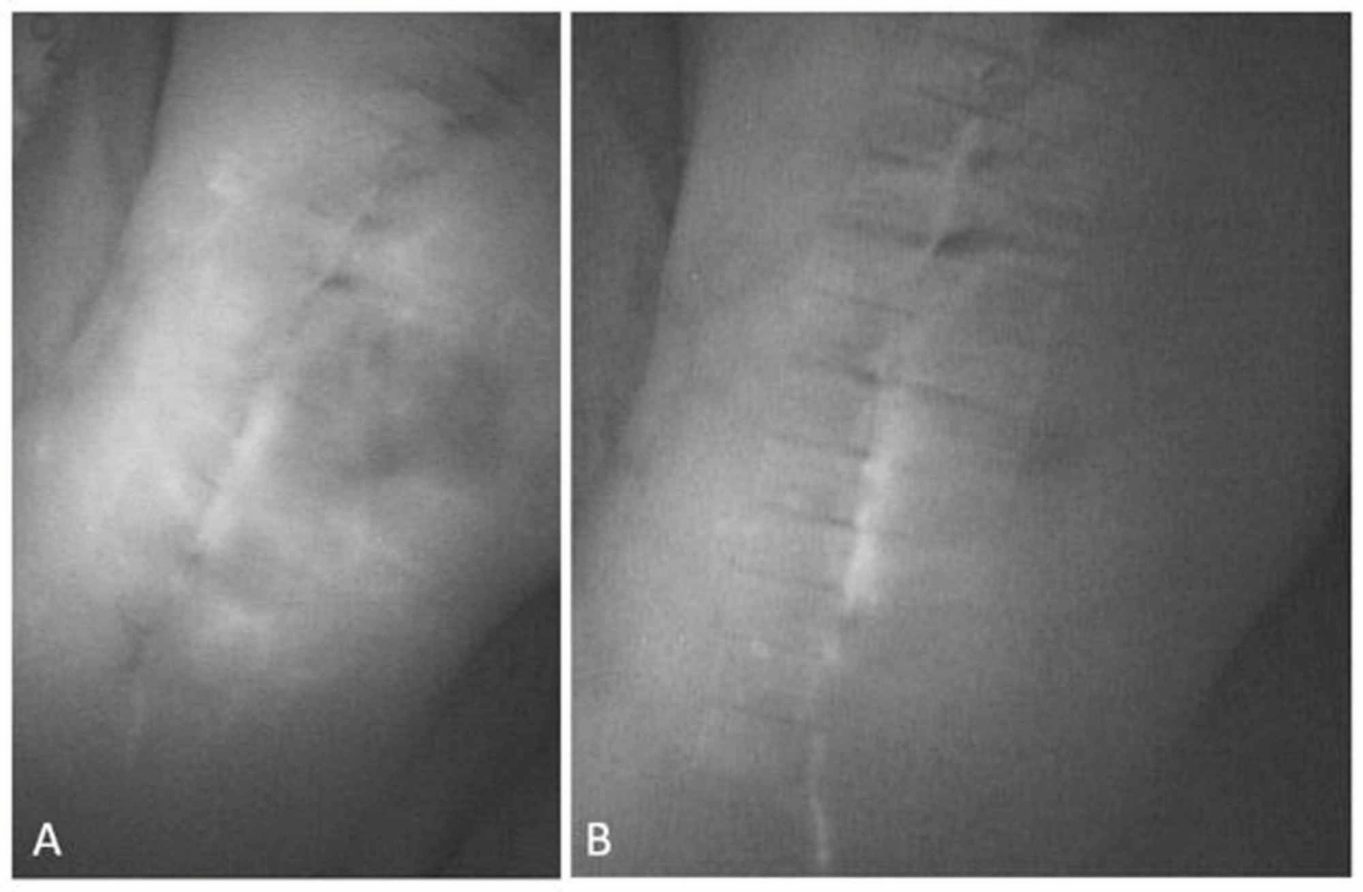
A baseline image of the blood supply to the knee (A), followed by five minutes of icing to the posterior aspect of the knee (B) Note the reduced blood flow following icing compared to the baseline image.

Examples of baseline images and images obtained immediately after icing the knee are shown in Figures 1 and 2, for incisional and posterior icing, respectively.

**Figure 3:**
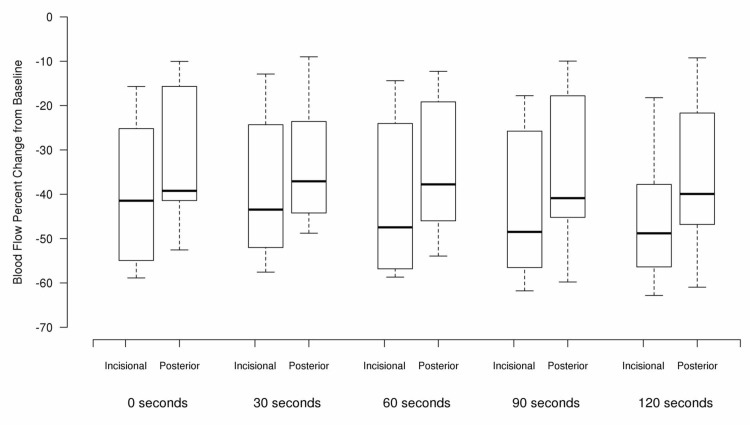
Percentage change in blood flow from baseline for incisional and posterior areas of the knee

As can be seen in Figures 1 and 2, there was a decrease in blood flow following icing both the incision as well as posteriorly. These changes are summarized for all patients in Table 1 and Figure 3 for incisional and posterior icing. Compared to baseline, blood flow was significantly lower at all post-baseline time points for both the incisional and posterior areas of the knee (*p* ≤ 0.004).

**Table 1:** Percent change in blood flow from baseline for incisional and posterior icing of the knee after TKA The sample median (range) is given to summarize the percentage change in blood flow from baseline as well as the difference in percentage change in blood flow from baseline between posterior and incisional areas. *P*-values vs. baseline for the separate incisional and posterior areas result from one-sample Wilcoxon rank sum tests, where the percentage change values were compared to a value of zero. *P*-values for comparisons of percentage blood flow from baseline result from paired Wilcoxon signed rank tests. One patient was missing value for 120-second blood flow in the incisional area. TKA, total knee arthroplasty

	Incisional		Posterior		Posterior vs. Incisional	
Time point	Percentage change in blood flow from baseline	P-value vs. baseline	Percentage change in blood flow from baseline	P-value vs. baseline	Difference in percentage change from baseline (incisional minus posterior)	P-value
0 seconds	-41.5 (-58.9, -15.7)	0.002	-39.2 (-52.6, -10.0)	0.002	-9.8 (-16.5, 0.0)	0.002
30 seconds	-43.5 (-57.6, -12.9)	0.002	-37.1 (-48.8, -9.0)	0.002	-7.4 (-12.0, 7.0)	0.014
60 seconds	-47.5 (-58.7, -14.4)	0.002	-37.8 (-53.9, -12.3)	0.002	-7.0 (-15.4, 4.8)	0.014
90 seconds	-48.5 (-61.8, -17.8)	0.002	-40.9 (-59.8, -10.0)	0.002	-8.0 (-17.1, 6.1)	0.020
120 seconds	-48.8 (-62.8, -18.2)	0.004	-39.9 (-61.0, -9.2)	0.002	-6.7 (-13.1, 3.5)	0.020

When considering each pair of sequential time points starting at time 0 (after removal of the ice), there were no significant differences in percentage change in blood flow from one time point to the following time point (i.e. 0 to 30 seconds, 30 to 60 seconds, etc.) for either incisional and posterior treatments (all *p* ≥ 0.084). There was a greater decrease in blood flow compared to baseline for the incisional area compared to the posterior area for all time points (all *p* ≤ 0.020).

## Discussion

Cryotherapy involves cooling the tissue, and this can be performed simply with ice or gel packs or with expensive cooling units. Multiple studies have demonstrated the potential benefits of cryotherapy on injured tissue. These benefits are reported to include reducing the permeability of capillaries and subsequent invasion of white blood cells into the local environment [3,10-11]. It is also believed that cryotherapy reduces the metabolic rate of cells, which is thought to provide a protective mechanism for at-risk cells in the ischemic injury environment. This was shown in an experiment by Merrick et al., who demonstrated that continuous cryotherapy inhibited the loss of mitochondrial oxidative function following a crush injury [12].

In addition to the benefits of cryotherapy at the cellular level, several studies have demonstrated improved outcomes after knee and hip surgery. In a prospective randomized study by Bjorn et al., they found that using cryotherapy helped reduce hospital length of stay in their patients [13]. Additional studies have found decreased blood loss, improved functional scores, improved pain relief, and reduced narcotic consumption with the use of cryotherapy postoperatively [14]. Two studies, however, provide conflicting results and report no difference in outcome parameters, including the length of stay and narcotic consumption with postoperative cryotherapy use [15-16].

Despite the reported benefits of cryotherapy, there are risks associated with use. Algafly demonstrated reduced nerve conduction velocities with the use of cryotherapy [17]. Several case reports have published on peripheral nerve injuries following prolonged use of cryotherapy [18-19]. Most cases resolved spontaneously but took several weeks to do so. Following their report, Drez et al. recommended using cryotherapy for no more than 30 minutes at a time to reduce the risk of complications [20].

In addition to nerve palsies, other studies have reported on frostbite injuries and severe wound complications following prolonged use [21-24]. Several of these authors have recommended that cryotherapy be limited in duration and even avoided in sedated or uncooperative patients and anesthetized extremities [21-22].

It is believed that nerve injuries and wound complications from prolonged cryotherapy are due to a non-freezing cold injury, similar to frostbite. In 1985, Francis reported that vasoconstriction was central to the development of a non-freezing cold injury [25]. Research since then has demonstrated sustained vasoconstriction following cryotherapy treatment that remained reduced long after the skin rewarmed to room temperature (a hysteresis loop) [4-5]. Khoshnevis warned that the depressed blood flow might place the tissue at risk for a non-freezing cold injury [5]. These previous studies, however, have evaluated the skin blood flow using indirect means of measurement. In our study, with laser-induced fluorescence angiography, we were able to directly visualize the skin blood flow.

Laser-induced fluorescence angiography with indocyanine green has been well-studied in the plastic and general surgery literature [8,9]. It has been used to identify sentinel lymph nodes, to document lymphatic pathways in lymphedema, and to assess flap viability [26]. For general surgery, Indocyanine green has been used to assess intraoperative perfusion in abdominal hernia repairs and to evaluate soft tissue viability and flap reconstruction in war-related trauma [27-28].

Intraoperative evaluation of skin-flap viability using laser-induced fluorescence with indocyanine green was first documented by Holm et al. They found that tissue-filling defects correlated with wound complications in 50% of the patients and that patients without filling defects healed uneventfully [29]. More recently this technique has been used in orthopedics in the evaluation of complex total knee arthroplasty closures. The authors of the study reported that this tool may help to identify at-risk wounds [7]. 

In our study, we used this technique to compare blood flow to the peri-incisional skin at baseline and again after applying ice to either the incisional area or the posterior aspect of the knee. We hypothesized that there would be a greater reduction in skin blood flow when the ice was placed over the incision due to direct cooling and vasoconstriction of this skin. We observed a statistically significantly greater than 40% decrease in the skin blood flow when ice was placed on the incision. 

We additionally hypothesized that placing ice on the posterior aspect of the knee would result in less of a decrease in peri-incisional skin blood flow compared to placing ice on the incision. We theorized that the ice would have less of a vasoconstrictive effect on the larger geniculate vessels supplying blood flow to the skin than if the ice was placed over the incision and directly affecting the smaller perforating vessels. We found that the decrease in blood flow compared to baseline was statistically less than that observed when ice was placed on the incision. However, as differences in blood flow reduction between incisional and posterior areas were only between 5% and 10%, these are likely not clinically significant differences.

In addition to the initial decrease in blood flow, we observed a sustained drop over the ensuing two minutes after removal of the ice. We did not find a significant change in the blood flow during this period for either the incisional or posterior icing groups. This finding supports previous research which demonstrated prolonged vasoconstriction following cryotherapy [4-5].

There are several limitations to this study. The main limitation was the small sample size. Though we were able to identify statistically significant differences in blood flow, larger studies are needed to more thoroughly evaluate the effect of icing the knee on blood flow following TKA. Another limitation is the possible carry-over effect from first icing the incision to icing the posterior knee. Although our study allowed for the skin to rewarm to room temperature between incisional and posterior icing and we re-dosed the ICG dye to obtain a new baseline for the posterior icing, it is possible that the vessels were already in a vasoconstricted state from the incisional icing (hysteresis effect) and therefore had less ability to vasoconstrict further during posterior icing. Finally, there were limitations to the technology. Several imaging systems are available and some provide features such as real-time reporting of fluorescent units while other devices only provide an image and the user relies on image software to report values at a later date. There is no current fluorescent unit threshold under which healing is thought to be impaired [30]. Previous researchers have used the technology to simply observe for filling defects rather than absolute numbers to indicate healing potential, and have reported success with this approach [29].

## Conclusions

In summary, we found a statistically significant decrease in peri-incisional blood flow with both anterior (incisional) and posterior icing of the knee. Physicians should be cognizant of this when recommending cryotherapy to patients after surgery, especially in at-risk wounds.
